# Distribution of α7 Nicotinic Acetylcholine Receptor Subunit mRNA in the Developing Mouse

**DOI:** 10.3389/fnana.2019.00076

**Published:** 2019-08-06

**Authors:** Ron S. Broide, Ursula H. Winzer-Serhan, Yling Chen, Frances M. Leslie

**Affiliations:** ^1^Department of Pharmacology, University of California, Irvine, Irvine, CA, United States; ^2^Department of Neuroscience and Experimental Therapeutics, Texas A&M University College of Medicine, Bryan, TX, United States

**Keywords:** cortex, hippocampus, spinal cord, enteric nervous system, adrenal medulla, kidney, dorsal root ganglia, nicotine

## Abstract

Homomeric α7 nicotinic acetylcholine receptors (nAChRs) are abundantly expressed in the central and peripheral nervous system (CNS and PNS, respectively), and spinal cord. In addition, expression and functional responses have been reported in non-neuronal tissue. In the nervous system, α7 nAChR subunit expression appears early during embryonic development and is often transiently upregulated, but little is known about their prenatal expression outside of the nervous system. For understanding potential short-term and long-term effects of gestational nicotine exposure, it is important to know the temporal and spatial expression of α7 nAChRs throughout the body. To that end, we studied the expression of α7 nAChR subunit mRNA using highly sensitive isotopic *in situ* hybridization in embryonic and neonatal whole-body mouse sections starting at gestational day 13. The results revealed expression of α7 mRNA as early as embryonic day 13 in the PNS, including dorsal root ganglia, parasympathetic and sympathetic ganglia, with the strongest expression in the superior cervical ganglion, and low to moderate levels were detected in brain and spinal cord, respectively, which rapidly increased in intensity with embryonic age. In addition, robust α7 mRNA expression was detected in the adrenal medulla, and low to moderate expression in selected peripheral tissues during embryonic development, potentially related to cells derived from the neural crest. Little or no mRNA expression was detected in thymus or spleen, sites of immune cell maturation. The results suggest that prenatal nicotine exposure could potentially affect the nervous system with limited effects in non-neural tissues.

## Introduction

Nicotinic acetylcholine receptors (nAChRs) are ligand gated pentameric cation channels, which were first identified in Torpedo electric organ, and are found in vertebrate and non-vertebrate animals (Changeux, 2012). The nAChRs can be broadly classified into muscle and neuronal type nAChRs, based on the compositions of the subunits that form the ion channel. The neuronal type nAChRs exhibit great diversity, and heteromeric receptors are formed by various compositions of alpha (α2–α10) and non-alpha (β2–β4) subunits. In addition, the α7 subunit forms homomeric pentamers which have high permeability for calcium ions (Papke, 2014; Kabbani and Nichols, 2018).

Neuronal nAChRs are widely distributed in the peripheral (PNS), central (CNS), and enteric (ENS) nervous system, sensory neurons, retina, and in the adrenal medulla. In the brain and spinal cord, α4 and β2 subunits are broadly expressed, and form the widely distributed neuronal heteromeric nAChR that displays high affinity for nicotine, and is primarily located on presynaptic terminals (Papke, 2014). Knock-out of either α4 or β2 mRNAs results in an almost complete loss of high affinity nicotine binding sites in the brain (Baddick and Marks, 2011). Homomeric α7 nAChRs, which are distinguished from neuronal heteromeric nAChRs by their high-affinity binding to α-Bungarotoxin (α-BTX), are also abundantly expressed in the CNS and spinal cord, where they are located at pre-and postsynaptic sites (Tribollet et al., 2004). During development, mRNA expression of several subunits is transiently upregulated, both in terms of intensity and spatial distribution, and different nAChR subtypes may take on specific functions relevant for brain development (Winzer-Serhan and Leslie, 1997, 2005; Broide and Leslie, 1999; Adams et al., 2002; Son and Winzer-Serhan, 2006). In the PNS, nAChRs exert essential functions in the regulation of the sympathetic and parasympathetic nervous systems. Moreover, neuronal nAChRs are expressed at high levels in peripheral ganglia, where heteromeric nAChRs are predominantly found (Wang et al., 2002). Homomeric α7 nAChRs are also detected in the periphery, but their functional role in either the PNS or ENS, particularly during development, is still unclear.

For a long time, the consensus was that neuronal nAChRs are expressed exclusively on neurons. However, in recent years it has become increasingly clear that functional nAChR responses can be found in non-excitable cells, including microglia (Shytle et al., 2004; Suzuki et al., 2006), astrocytes (Papouin et al., 2017), Schwann cells (Petrov et al., 2014), and other non-neuronal tissues (Sharma and Vijayaraghavan, 2002), and that these responses are often mediated by α7 nAChRs. Of particular interest is the expression of α7 nAChRs in the immune system, because of its crucial role in the regulation of the cholinergic anti-inflammatory pathway (Wang et al., 2003). Based on findings from previous studies, neuronal nAChR subunits appear early during brain development (Zoli et al., 1995; Adams et al., 2002; Tribollet et al., 2004). However, at present, little is known about the expression of α7 nAChRs outside of the CNS, especially during embryonic development. To better understand the potential developmental roles of α7 nAChRs, and the possible short-term and long-term effects of gestational nicotine exposure, we used highly sensitive isotopic *in situ* hybridization (Winzer-Serhan et al., 1999) to identify α7 nAChR expression in embryonic and neonatal whole-body mouse sections. The results of this study revealed the expression of α7 mRNA as early as embryonic day 13 in the peripheral nervous system, including dorsal root ganglia, parasympathetic and sympathetic ganglia, followed by strong expression in brain and spinal cord. In addition, α7 subunit mRNA expression was detected in a number of peripheral tissues potentially related to cells derived from the neural crest. In contrast, little or no expression was detected in thymus or spleen, sites of immune cell maturation.

## Materials and Methods

The following materials were obtained from the sources indicated: bovine serum albumin, ficoll, polyvinylpyrrolidone, poly-L-lysine, and RNase A (Sigma Chemical Co., St. Louis, MO, United States); T3 and T7 RNA polymerases, proteinase K, and yeast tRNA (Roche Molecular); formamide (Fluka, Ronkonkoma, NY, United States); dextran sulfate and Hyperfilm Bmax (Amersham Pharmacia, Arlington Heights, IL, United States); nuclear track emulsion (NTB-2), (Kodak, Rochester, NY, United States); [^35^S]uridine triphosphate (UTP) (Life Science NEN, Boston, MA, United States).

### Tissue Preparation

Male and female mice (C57BL/6; Charles River, Wilmington, MA, United States) were group housed and provided with food and water *ad libitum*. The mice were mated over a 4-day period and females were monitored twice daily (morning and afternoon) for vaginal plugs. The day of mating (presence of a plug) was defined as embryonic day (E) 0 and pups were born on E19. Pregnant female mice were killed by decapitation and their embryos removed at E13, E15, and E17. Select embryonic pups were taken from different litters and their sex was identified whenever possible. Embryonic and postnatal day (P) 0 mice were anesthetized on ice for several minutes before being frozen whole in isopentane at –25°C and stored at −80°C until use. These procedures were approved by the Institutional Animal Care and Use Committee at the University of California, Irvine, in accordance with federal guidelines. Mouse pups (*n* = 2–3/age) were cryostat sectioned (20 μm) in the sagittal plane from the lateral right side of the body until just past the midline. Tissue sections were mounted onto slides which were coated with either 1% gelatin (for histology) or an additional coating of poly-L-lysine (for *in situ* hybridization) and kept at –20°C. Sections were post-fixed with 4% paraformaldehyde in 0.1 M phosphate buffered saline (PBS), pH 7.4 for 1 h at 22°C, then washed in PBS, air dried and stored desiccated at −20°C until use.

### cRNA Probe Preparation and *in situ* Hybridization

cRNA riboprobes labeled with [^35^S]UTP were prepared from a 279 bp *Pst*I restriction DNA fragment encoding the third intracellular loop of the mouse α7 nAChR subunit (NM_007390.3; nucleotide sequence 1140 to 1419) (Orr-Urtreger et al., 1995). Riboprobes targeted to this region have been used previously to characterize α7 mRNA distribution in developing mice (Orr-Urtreger et al., 2000; Broide et al., 2001) and show the same pattern of expression as those observed using riboprobes targeted to either the 3′ non-coding region or the full-length mouse α7 nAChR cDNA (data not shown). Post-fixed whole-body sections were processed according to a published protocol (Winzer-Serhan et al., 1999). Briefly, slide-mounted sections were first preincubated with 0.1 μg/ml proteinase K for 10 min at 22°C, and then incubated for 18 h at 60°C with a hybridization solution (50% formamide, 10% dextran sulfate, 0.02% Ficoll, 0.02% polyvinyl pyrrolidone, 0.02% bovine serum albumin, 500 μg/ml tRNA, 10 mM dithiothreitol, 0.3 M NaCl, 10 mM Tris, pH 8.0, 1 mM EDTA, pH 8.0) containing [^35^S]UTP-labeled cRNA riboprobes (1 × 10^7^ cpm/ml) in the antisense orientation. Adjacent sections were incubated with riboprobes in the sense orientation to define non-specific hybridization. Tissue sections were then incubated with RNase A (20 μg/ml) for 30 min at 37°C, followed by high-stringency washes of decreasing salinity in SSC (sodium chloride/sodium citrate) buffer, and a 30 min wash in 0.1 × SSC at 60°C. Sections were dehydrated, apposed to β-max film for 3–6 days at 4°C, and then dipped in liquid NTB-2 emulsion. Following a 2-week exposure, slide were developed in Kodak D-19, fixed, counter-stained with Cresyl-Violet, cover-slipped and analyzed by high-powered microscopy.

### Data Analysis

An initial anatomical analysis was undertaken in which autoradiographic images on β-max films were compared to their corresponding Nissl-stained sections, at each developmental age. Emulsion dipped slides were then analyzed by microscopy to more accurately define areas expressing α7 mRNA. Labeled anatomic regions of embryonic and postnatal mouse bodies were identified using several different sources (Kaufman, 1995; Jacobowitz and Abbott, 1998; Kaufman and Bard, 1999; Paxinos et al., 2007). Silver grains in these regions were manually counted at 400× magnification within a 2 × 2 grid area encompassing 50 μm^2^ and the results were expressed as the average number of silver grains per single (25 μm^2^) grid box. Based on these counts, a score was assigned to the labeled anatomical structure: + (>10 grains/25 μm^2^), ++ (>20 grains/25 μm^2^), +++ (>50 grains/25 μm^2^), ++++ (>90 grains/25 μm^2^). In some regions, a double quantitative score was ascribed (e.g., +/+++), which signified lower levels of mRNA surrounding spots of more intense signal. Background labeling (<10 grains/25 μm^2^) was established as the density of silver grains in non-neural tissues with high cellular density (such as the liver), or with high density of extracellular matrix (such as cartilage), or the density of grains over neural structures after hybridization with the sense riboprobe.

## Results

**FIGURE 1:**
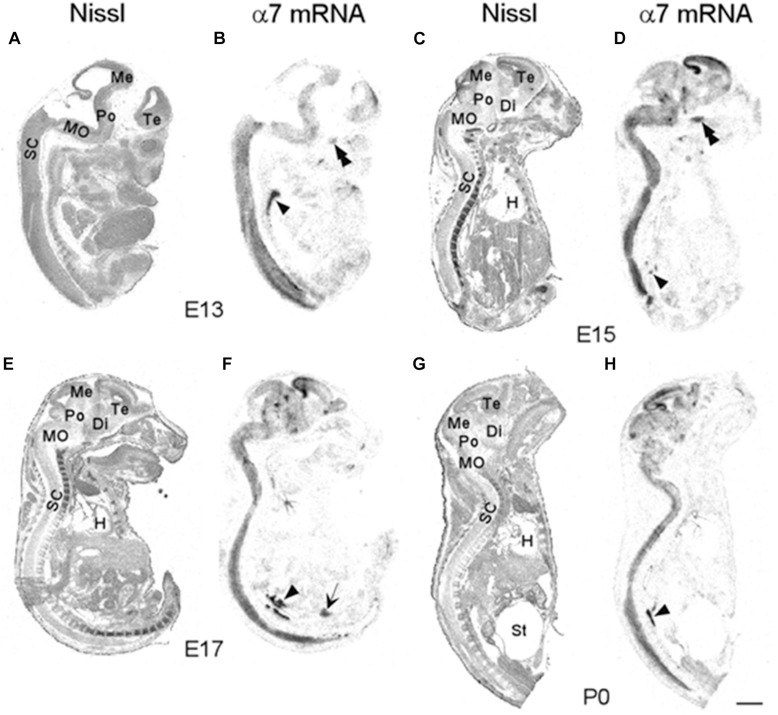
Bright-field micrographs of autoradiographic images showing α7 nAChR mRNA expression in sagittal midline sections of E13 **(B)**, E15 **(D)**, E17 **(F)**, and P0 **(H)** mouse pups. **(A,C,E,G)** Adjacent sections stained with cresyl-violet. Strong mRNA labeling can be seen in the autonomic (arrowhead) and trigeminal (double arrowheads) ganglia as well as the prostate (arrow). Di, diencephalon; H, heart; Me, mesencephalon; MO, medulla oblongata; Po, pons; SC, spinal chord; St, stomach; Te, telencephalon. Scale bar = 1 mm **(A,B)**, 1.5 mm **(C,D)**, 1.7 mm **(E,F)**, and 2 mm **(G,H)**.

We began our analysis at E13 because it was previously demonstrated that at this age, low levels of α7 nAChR mRNA expression first appear in several regions of the rat brain (Broide et al., 1995; Adams et al., 2002). However, because our present analysis of the mouse was more broad-based, encompassing the entire body, we conducted a more general investigation of the CNS with greater emphasis on regions showing higher α7 mRNA expression. An initial examination of autoradiographic images revealed that α7 nAChR mRNA was expressed throughout the CNS, including the spinal cord and retina, as early as E13, and up until birth (Figure 1). High levels of mRNA transcripts were also found in many PNS and sensory ganglia, and relatively low levels of α7 mRNA labeling were detected in some non-neuronal tissues during development. In general, at E13, α7 nAChR mRNA expression intensity was low to moderate in the PNS and CNS, and low in non-neuronal areas of the body (Figure 1 and Tables 1–3). However, by E15, there was a marked increase in mRNA expression intensity in areas of the nervous system that persisted until birth, whereas expression in non-neuronal areas remained low.

**TABLE 1:** α7 nAChR subunit mRNA expression in the developing central nervous system (CNS).

		**Age**		
**Region**	**E13**	**E15**	**E17**	**P0**
Olfactory bulb	–	++	++	++
Olfactory tubercle	–	+++	++++	+++
**Telencephalon**				
Parietal Cortex				
Cortical plate	+++	++++	++++	++++
Layer 6	NA	NA	++++	+++
Intermediate zone	NA	++	++	+
Ventricular zone	–	+	+	–
Hippo. formation	++	++++	++++	+++/++++
Septum	–	–	+	+
Basal ganglia	+	+	+/++	+/++
Preoptic area	++	++	++	++
**Diencephalon**				
Thalamus				
Lateral	–/+	+/+++	+/++++	+/+++
Medial	–/+	+/++	+/+++	+/+++
Hypothalamus	–/+	+/++	+/+++	+/+++
**Mesencephalon**				
Lateral	–	+	+/++	+/++
Mediodorsal	+	+	+	++
Medioventral	–	+/++	+/++	+/++
**Pons**				
Lateral	+	+/++	+/++	+/++
Mediodorsal	+/+++	+/++++	+/++++	+/++++
Medioventral	+	+/+++	+/+++	+/+++
Medulla oblongata				
Lateral	+	++	++	++
Mediodorsal	+/++	++/+++	++/+++	++/+++
Medioventral	+/++	++/++++	++/++++	++/+++
Cerebellum	NA	+	+	+
**Spinal chord**				
Dorsal	++	+++	+++	+++
Ventral	++/+++	+++/++++	+++/++++	+++/++++

*Intensity is based on silver grain counts: + (>10 grains/25 μm^2^), ++ (>20 grains/25 μm^2^), +++ (>50 grains/25 μm^2^), ++++ (>90 grains/25 μm^2^). A double quantitative score indicates signified lower levels of mRNA surrounding spots of more intense signal.*

**TABLE 2:** α7 nAChR subunit mRNA expression in ganglia of the developing peripheral nervous system (PNS).

		**Age**		
**Ganglion**	**E13**	**E15**	**E17**	**P0**
***Sensory***				
Trigeminal (V)	++	+++/++++	+++/++++	+++/++++
Dorsal root	+	+++/++++	+++/++++	+++/++++
**Autonomic ganglia**				
***Sympathetic***				
Superior cervical	+++	++++	++++	++++
Stellate	+++	++++	++++	++++
Celiac	+++	++++	++++	++++
Mesenteric	+++	++++	++++	++++
***Parasympathetic***				
Submandibular (VII)	+++	+++	+++	+++
Otic (IX)	+++	++++	++++	++++
Vagal (X)	+/++	++/++++	++/++++	++/++++
Intracardiac	+++	+++	+++	+++
Intramural	+++	++++	++++	+++
Enteric	+	+++	+++	++/+++

*Intensity is based on silver grain counts: + (>10 grains/25 μm^2^), ++ (>20 grains/25 μm^2^), +++ (>50 grains/25 μm^2^), and ++++ (>90 grains/25 μm^2^). A double quantitative score indicates signified lower levels of mRNA surrounding spots of more intense signal.*

**TABLE 3:** Expression of α7 nAChR subunit mRNA in developing non-neuronal tissue.

		**Age**		
**Tissue**	**E13**	**E15**	**E17**	**P0**
Adrenal medulla	++	+++	+++	+++/++++
Kidney cortex	–/+	–/+++	–/+++	–/++
Tongue	–	–/+++	–/+++	–/++++
Nasal epithelium	–	+	+	+
Tooth bud	–	+	++	+
Aorta	++	++	++	++
Muscle	+/+++	+/++	+	–

*Intensity is based on silver grain counts: + (>10 grains/25 μm^2^), ++ (>20 grains/25 μm^2^), +++ (>50 grains/25 μm^2^), and ++++ (>90 grains/25 μm^2^). A double quantitative score indicates signified lower levels of mRNA surrounding spots of more intense signal.*

### Central Nervous System

#### Olfactory Bulb

**FIGURE 2:**
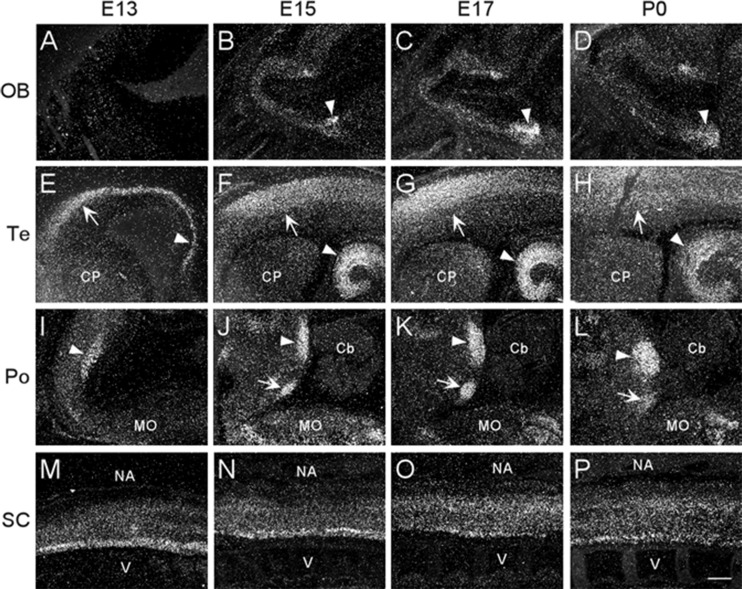
Dark-field photomicrographs showing α7 mRNA distribution in different regions of the CNS from parasagittal sections of E13, E15, E17, and P0 mouse pups. **(A–D)** In the area of the olfactory bulb (OB), arrowheads point to increasing mRNA expression in the olfactory tubercle. **(E–H)** In the telencephalon (Te), strong mRNA expression is observed in the parietal cortex (arrow) and hippocampus (arrowhead). **(I–L)** In the pons, the mRNA expression is highest in the dorsal tegmental (arrowhead) and ventral tegmental (arrow) nuclei. **(M–P)** In the spinal cord (SC), a band of elevated mRNA expression can be seen in the ventral, motor neuron region. Cb, cerebellum; CP, caudate putamen; MO, medulla oblongata; NA, neural arch; V, vertebrate. Scale bar = 300 μm.

At E13, emulsion dipped, sagittal whole-body sections containing the emerging olfactory bulb showed background labeling in this region (Figure 2A). By E15, low levels of α7 nAChR mRNA expression were first observed in the olfactory bulb and were visible throughout embryonic development (Figures 2B,D and Table 1). The olfactory tubercle exhibited higher levels of mRNA expression that peaked slightly at E17 (Table 1).

#### Telencephalon

In the developing mouse telencephalon, α7 nAChR mRNA was most prominently expressed in the neocortex and the hippocampus (Figure 2 and Table 1). At E13, moderate levels of α7 mRNA were already observed throughout the cortical plate and the emerging hippocampal formation (Figure 2E). Levels of α7 mRNA in these regions were increased by E15 and remained high until birth (Figures 2F–H). In the neocortex, high levels of α7 mRNA expression were largely restricted to the parietal cortex. Elevated levels of mRNA were also expressed in the emerging layer 6 of the cortex, beginning at E17 (Table 1). Lower levels of mRNA labeling were observed in the intermediate and ventricular zones, as well as the basal ganglia, preoptic area, and the septum (Table 1).

#### Diencephalon

Levels of α7 nAChR mRNA in the diencephalon at E13 were extremely low (Table 1). However, by E15, mRNA levels were increased in various nuclei throughout the thalamus and hypothalamus, and remained at moderate levels until birth.

#### Mesencephalon

The developing mesencephalon is comprised of the superior and inferior colliculi at its dorsal subdivision and the tegmentum, including the red nucleus in the ventral subdivision. At E13, mostly background labeling was observed in this region of the brain (Table 1). Levels of α7 mRNA in the mesencephalon showed a slight increase by E15 and remained relatively low throughout embryonic development. However, higher levels of mRNA were particularly notable in the red nucleus and the superior and inferior colliculi (data not shown).

#### Pons

The region of the pons that contains the locus coeruleus (LC) and dorsal raphe (DR), showed strong labeling for α7 nAChR mRNA in the dorsal tegmental and ventral tegmental nuclei from E13 until birth (Figures 2I–L). Low to moderate levels of mRNA were found throughout the rest of the pons (Table 1).

#### Medulla Oblongata

At E13, low levels of α7 nAChR mRNA expression were observed in the medulla oblongata. However, by E15, levels of mRNA were increased, particularly in the ventral region, and remained elevated until birth (Table 1). The mRNA expression pattern was mainly scattered with some dense areas of labeling in both the dorsal and ventral regions (Figures 2I–L).

#### Cerebellum

The cerebellum, which was first visible by E15, showed very low levels of α7 mRNA expression throughout embryonic development (Figures 2I–L and Table 1).

#### Spinal Cord

Moderate levels of α7 mRNA expression were visible in the spinal cord (SC) at E13 and remained relatively unchanged until birth (Figures 2M–P and Table 1). In the dorsal region of the SC, the expression of α7 mRNA was homogenous. However, in the ventral region, a band of increased α7 mRNA expression was observed with areas of intense cellular labeling (Figures 2M–P).

#### Retina

**FIGURE 3:**
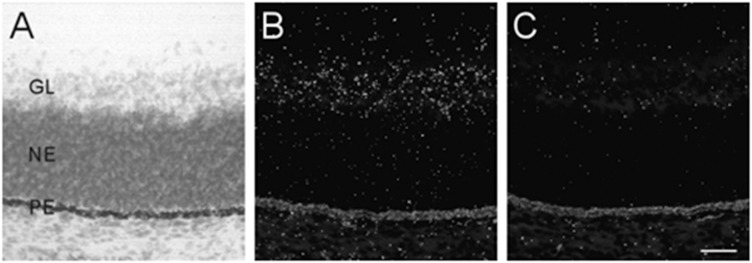
α7 nAChR mRNA distribution in the developing retina. **(A,B)** Bright- and dark-field photomicrographs of an E17 retina showing low levels of α7 mRNA expression in the ganglion cell layer. The section was counterstained with cresyl-violet and retinal layers are indicated: GL, ganglion cell layer; NE, neural epithelium; PE, pigment epithelium. **(C)** Adjacent section showing background labeling with the sense riboprobe. Scale bar = 50 μm.

The developing retina exhibited background labeling at E13. By E15, low levels of α7 mRNA expression became detectable in the emerging ganglionic cell layer. Figure 3 shows an example of α7 mRNA labeling in an E17 retina. This level of scattered labeling, which was not found in adjacent sections hybridized with the sense riboprobe (Figure 3C), was observed throughout embryonic development and at birth.

### Peripheral Nervous System

α7 nAChR mRNA was expressed in all discernible ganglia of the developing mouse PNS. However, due to technical issues in gathering tissue sections, not all ganglia were observed at every age analyzed. Therefore, the representative ganglia that are discussed are those that were visible throughout embryonic development.

#### Sensory Ganglia

**FIGURE 4:**
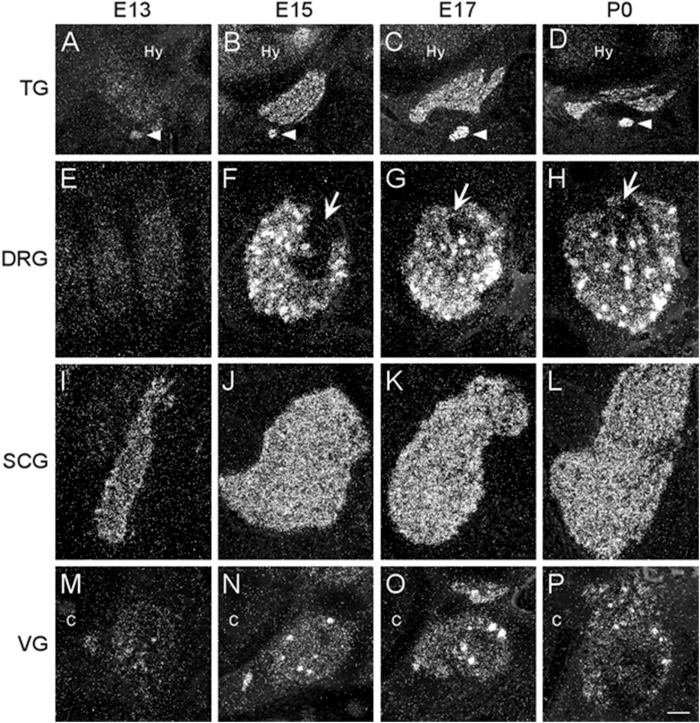
Dark-field photomicrographs of representative sensory and autonomic ganglia at E13, E15, E17, and P0. **(A–D)** Increasing α7 mRNA expression is observed with age in the trigeminal sensory ganglion (TG) and the optic parasympathetic ganglion (arrowhead). **(E–H)** α7 mRNA expression in the dorsal root ganglion (DRG) is low at E13, but shows intense punctate expression starting at E15. The entry point for the dorsal root is indicated (arrows). **(I–L)** An even distribution of strong α7 mRNA expression is observed in the superior cervical ganglion (SCG), part of the chain of sympathetic ganglia along the spinal cord. **(M–P)** Expression of α7 mRNA in the vagal parasympathetic ganglion (VG) at the base of the brain. Hy, hypothalamus; C, cochlea. Space bar = 250 μm **(A–D)**, 100 μm **(E–P)**.

We examined two different sensory ganglia, the trigeminal and the dorsal root ganglion. The trigeminal ganglion (TG) is a sensory ganglion found at the base of the brain. Primary afferent fibers from this ganglion form much of the fifth cranial nerve (V), which innervates the face, jaw and olfactory mucosa. At E13, the TG exhibited low levels of α7 mRNA labeling (Figure 4A and Table 2). By E15, levels of mRNA were markedly increased and remained elevated until birth (Figures 4B–D and Table 2). The α7 mRNA expression pattern in the TG appeared striated with random cells expressing a higher density of mRNA transcripts.

The dorsal root ganglia (DRG) are attached to the dorsal horn on either side, and along the length of the spinal cord. They receive input from the various sensory receptors throughout the body and relay this information to the spinal cord. At E13, the DRG showed very low expression of α7 mRNA transcripts (Figure 4E and Table 2). The levels of mRNA were markedly increased by E15 and remained elevated until birth (Figures 4F–H and Table 2). The α7 mRNA expression pattern in the DRG was homogenous, with intermittent clusters of cells expressing a high density of mRNA transcripts.

The vagal (jugular) ganglion (VG), located just behind the cochlea, is a well-marked ganglionic enlargement of the vagus nerve (X) complex that provides afferent somatosensory innervation to the external auditory meatus, cranial meninges, and the tympanic membrane. At E13, the vagal ganglion showed very low levels of α7 mRNA expression (Figure 4M and Table 2). By E15, the overall levels of α7 mRNA were increased to moderate levels, and random cells were observed expressing very high mRNA levels. This pattern and level of expression persisted until birth (Figures 4N–P and Table 2).

#### Autonomic Sympathetic Ganglia

The sympathetic chain of ganglia are located next to the vertebral column and overlying the descending aorta. Together they provide sympathetic innervation to all the organs of the body. Although α7 nAChR mRNA labeling was observed throughout the sympathetic chain, we focused primarily on the larger, superior cervical ganglia (SCG), which are located at the top of this chain and innervate the radial muscle of the iris, the eyelids, the lacrimal and salivary glands, and the heart. At E13, the SCG exhibited moderate levels of α7 mRNA expression (Figure 4I and Table 2). However, by E15, the levels of α7 mRNA in the SCG were increased and remained high until birth (Figures 4J–L and Table 2). The α7 mRNA expression pattern was homogenous throughout the SCG. Similar spatiotemporal patterns of α7 mRNA expression were also observed in the stellate, celiac, and mesenteric sympathetic ganglia (Table 2).

#### Autonomic Parasympathetic Ganglia

The parasympathetic ganglia are located close to the organs they innervate and are often embedded within their walls. The cranial component of the parasympathetic system originates from four of the twelve cranial nerves that emerge from the brainstem. The glossopharyngeal nerve (IX) synapses onto neurons in the otic ganglion (OG) which innervates the parotid gland. Located just below the TG, the OG exhibited moderate levels of α7 mRNA expression at E13 (Figure 4A and Table 2). The levels of α7 mRNA were increased by E15 and remained high until birth (Figures 4B–D and Table 2).

**FIGURE 5:**
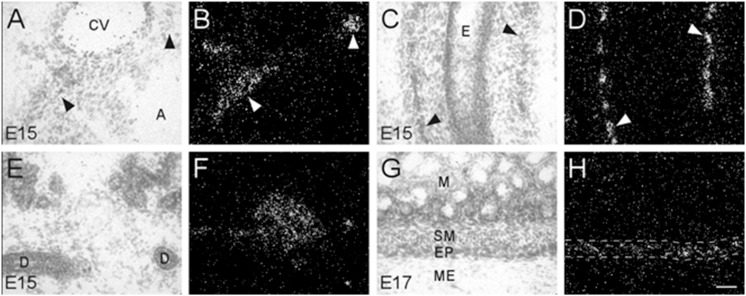
Bright- and dark-field photomicrographs showing α7 nAChR mRNA expression in various intramural ganglia of the parasympathetic system at E15 and E17. **(A,B)** α7 mRNA transcripts are expressed in intracardiac ganglia (arrowheads), that are interspersed on top of the atria. **(C,D)** Intramural ganglia (arrowheads) lining the esophagus **(E)** showing high levels of α7 mRNA expression. **(E,F)** Expression of α7 mRNA is observed in one of the submandibular ganglia within the submaxillary gland. **(G,H)** The enteric plexus of the large colon (area between dashed lines) shows light to moderate expression of α7 mRNA. Sections are counterstained with cresyl-violet. A, atrium; CV, cardiac vessel; D, submaxillary duct; E, esophagus; EP, enteric plexus; M, mucosa; ME, muscularis externa; SM, submucosa. Space bar = 50 μm.

During embryonic development, α7 nAChR mRNA transcripts were also detected in a number of parasympathetic ganglia embedded within different organs (Table 2). For example, α7 mRNA was expressed at moderate levels in intracardiac ganglia that are interspersed on top of the atria of the heart (Figures 5A,B). High levels of α7 mRNA expression were also found in intramural ganglia surrounding the esophagus, and were observed along its entire length (Figures 5C,D). Moderate levels of α7 mRNA expression were detected in submandibular ganglia which are embedded in the submaxillary gland (Figures 5E,F), and are innervated by the facial nerve (VII). Finally, ganglia in the enteric plexus of the gut displayed low to moderate levels of α7 mRNA expression during embryonic development. These ganglia were observed throughout the small and large intestine (Figures 5G,H).

### Non-neuronal Tissues

#### Adrenal Medulla and Kidney Cortex

**FIGURE 6:**
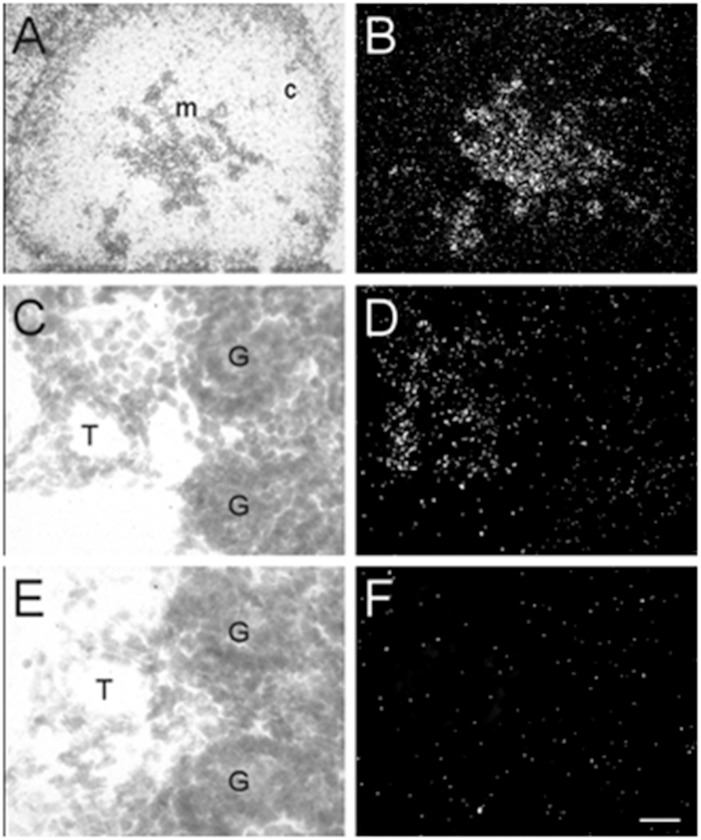
Bright- and dark-field photomicrographs of the adrenal gland and kidney cortex at E17 displaying α7 nAChR mRNA expression. **(A,B)** The adrenal medulla shows high levels of α7 mRNA expression. **(C,D)** In the kidney cortex, α7 mRNA is expressed mainly in tubules close to the glomeruli. **(E,F)** Adjacent section showing background labeling with the sense riboprobe in the kidney cortex. Sections are counterstained with cresyl-violet. C, adrenal cortex; G, glomerulus; M, adrenal medulla; T, kidney tubule. Scale bar = 100 mm **(A,B)**, 25 μm **(C–F)**.

In addition to the expression of α7 nAChR mRNA in the nervous system, α7 mRNA transcripts were also detected in a number of non-neuronal tissues throughout the bodies of embryonic and postnatal mice. At E13, low levels of α7 nAChR mRNA were detected in the adrenal medulla (Table 3). The mRNA levels increased by E15 and were highest at birth. The mRNA expression pattern delineated the adrenal medulla containing darkly stained chromaffin cells (Figures 6A,B). Low levels of α7 mRNA transcripts were observed in the kidney cortex at E13. The levels were notably increased by E15 followed by a slight decrease at birth (Table 3). The signal was detected in tubules proximal to the glomeruli (Figures 6C,D). Adjacent sections hybridized with the sense riboprobe showed only background levels over the tubules in the kidney cortex (Figures 6E,F).

#### Oral and Nasal Tissues

**FIGURE 7:**
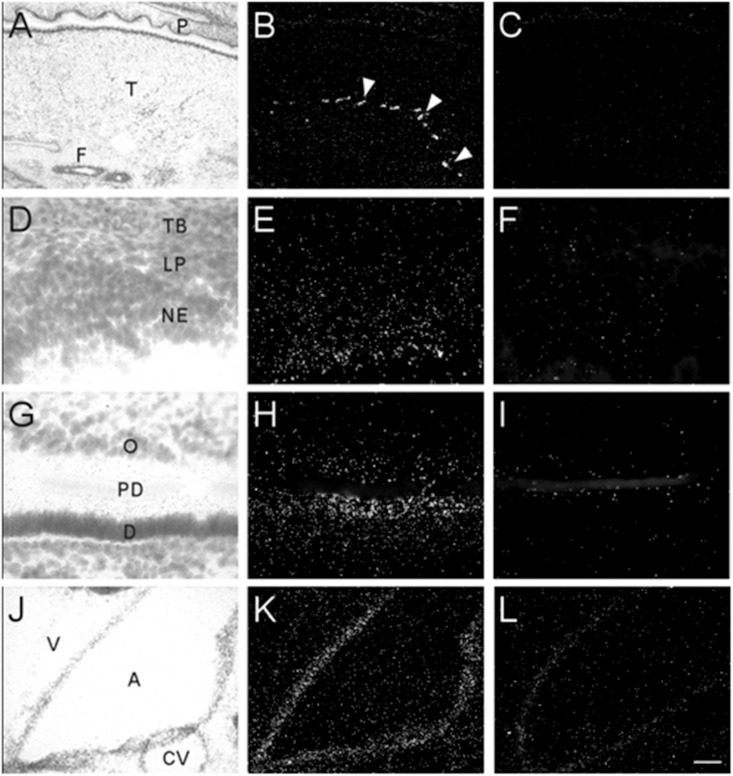
Bright- and dark-field photomicrographs showing α7 nAChR mRNA expression in various non-neuronal tissues from E17 and P0 mouse pups. **(A,B)** Strong punctate expression of α7mRNA in the posterior region of the tongue (arrow heads). **(D,E)** Low expression of α7mRNA is observed in the nasal epithelium. **(G,H)** Moderate levels α7mRNA expression are observed in the predentin layer of the developing tooth bud. **(J,K)** α7 mRNA expression is shown in epithelial cells lining the aorta. Sections are counterstained with cresyl-violet. **(C,F,I,L)** Adjacent sections showing background labeling in each tissue with the sense riboprobe. A, aorta; CV, cardiac vessel; D, dentine; F, frenulum; NE, nasal epithelium; O, odontoblasts; P, palate; PD, predentin; T, tongue; TB, turbinate bone; V, ventricle. Space bar = 250 μm **(A–C)**, 25 μm **(D–I)**, 100 μm **(J–L)**.

Transcripts for α7 nAChR mRNA were detected in several different non-neuronal tissues in the head region of embryonic and postnatal mice. These tissues all showed background labeling at E13 (Table 3). However, by E15, moderate levels of α7 mRNA expression were first observed in the posterior portion of the tongue, above the frenulum, and were increased by P0. The mRNA labeling exhibited a punctate chain pattern that was confined to the middle region of the tongue (Figures 7A,B). Very low levels of α7 mRNA labeling were also found in cells of the nasal epithelium beginning at E15 and up until birth (Table 3 and Figures 7D,E). Finally, low levels of α7 mRNA transcripts were first detected in the developing tooth bud at E15 and were markedly increased by birth (Table 3). The mRNA expression was highest in the predentin layer of the tooth bud, an area containing the extended cell bodies of the dentine-forming odontoblasts (Figures 7G,H). Expression of α7 was also detected in the cell line of the aorta (Figures 7J,K). Adjacent sections hybridized with the sense probe exhibited background levels of expression (Figures 7C,F,I,L).

#### Prostate and Testes

**FIGURE 8:**
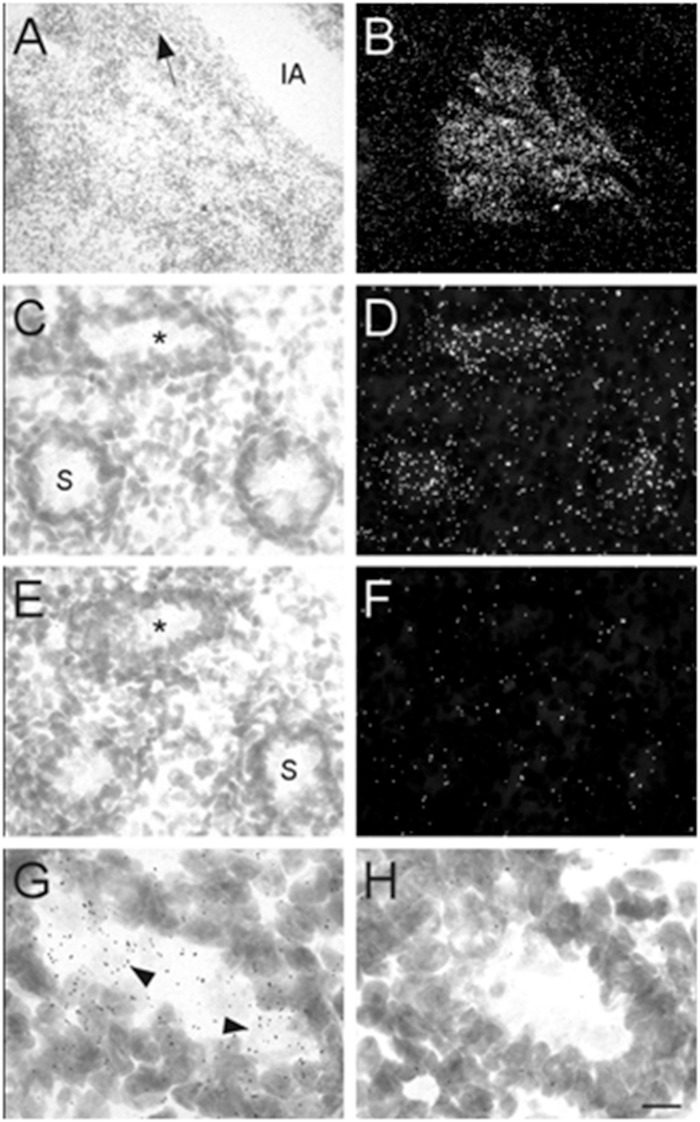
Bright- and dark-field photomicrographs showing α7 nAChR mRNA expression in the prostate and testes at E17. **(A,B)** High expression of α7 mRNA is observed in the mouse prostate, close to the iliac artery (IA). The arrow points toward the bladder. **(C,D)** α7 mRNA labeling can be seen within the seminiferous tubules (S) of the mouse testes. **(E,F)** Adjacent section showing background labeling with the sense riboprobe in the testes. **(G,H)** Higher magnification of tubules in **C** and **E** (asterisk) showing mRNA expression on cells within the seminiferous tubule. Sections are counterstained with cresyl-violet. Scale bar = 100 μm **(A,B)**; 25 μm **(C–F)**; 10 μm **(G,H)**.

Male mouse pups were only identified and obtained at E17 and P0. Therefore, our analysis of the prostate and testes was limited to these two ages. At E17, high levels of α7 nAChR mRNA expression were observed in the developing prostate (Figures 8A,B). This structure was located and identified just below the iliac artery and slightly dorsal of the bladder in the midline region. We also found low levels of α7 mRNA expression within the seminiferous tubules of the testes (Figures 8C,D). This labeling was not detected on adjacent sections hybridized with the sense riboprobe (Figures 8E,F). The α7 mRNA transcripts were expressed in the lumen of the tubules over cells with light Nissl staining (Figures 8G,H). Female embryos showed only background labeling over their ovaries (data not shown).

#### Muscle

**FIGURE 9:**
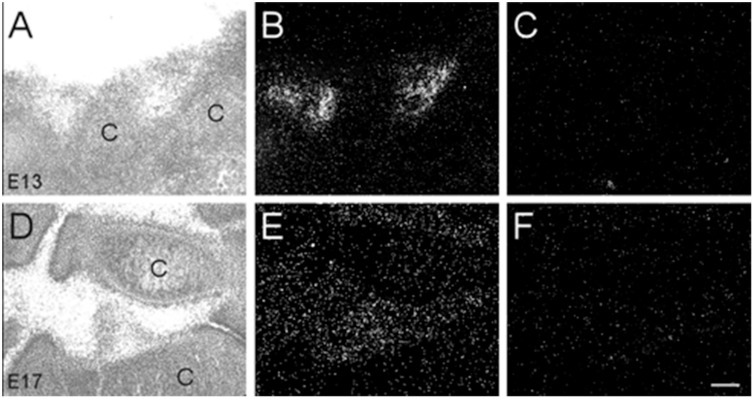
Bright- and dark-field photomicrographs showing α7 nAChR mRNA expression in muscle tissue at E13 and E17. **(A,B)** High expression of α7 mRNA is observed in the inter-digit regions of the limb buds at E13. **(D,E)** Lower expression of mRNA is observed in these regions at E17. **(C,F)** Adjacent sections showing background labeling with the sense riboprobe in the limb bud region. C, cartilage. Space bar = 100 μm.

Interestingly, we found α7 nAChR mRNA expression in developing muscle tissue. However, in contrast to the other body regions analyzed, the muscle showed a decrease in α7 mRNA expression during embryonic development (Table 3). Beginning at E13, low levels of α7 mRNA expression were observed over most of the muscle regions, with low to moderate expression levels exhibited in areas of the limbs close to the cartilage (Figures 9A,B). By E17, only low levels of mRNA were apparent over all muscle areas (Figures 9D,E) and by P0, these levels were the same as background (Table 3). Adjacent sections show hybridization signal with the sense probe (Figures 9C,F).

## Discussion

In the present study, we have demonstrated the widespread distribution of α7 nAChR mRNA throughout the embryonic mouse nervous systems, highlighting the ubiquitous expression of α7 mRNA in the central, peripheral and enteric nervous system during embryonic development. At E13, the youngest age examined, α7 nAChR mRNA expression was already moderately strong in most autonomic ganglia and spinal cord, and expression was also detected at lower levels in the medulla oblongata, pons and mesencephalon. After E13, α7 mRNA expression was generally increased, especially in autonomic and parasympathetic ganglia, and in cortical structures including the hippocampal anlage, and became more refined to specific brain structures and nuclei. These findings confirm previous studies in the rodent (Broide et al., 1995; Messi et al., 1997; Adams et al., 2002), chick (Erkman et al., 2000; Keiger et al., 2003), and human (Hellström-Lindahl et al., 1998; Falk et al., 2002) developing nervous system. Moreover, mRNA expression was observed in various non-neural tissues throughout the body, including adrenal medulla, kidney, tongue, tooth, nasal epithelium, prostate, testis and muscle, but with the exception of the adrenal medulla, expression levels were mostly low. This widespread expression of α7 nAChRs throughout the developing nervous system and the adrenal medulla suggest that these receptors might have important functions during development and that prenatal nicotine exposure may affect nervous system development via aberrant activation of α7 nAChRs (Broide and Leslie, 1999), which could particularly impact brain circuit formation (Lozada et al., 2012) and alter adrenal medulla function (Buttigieg et al., 2009). Additionally, there were low level of α7 mRNA expression in many tissues throughout the body. However, it remains to be determined if the transcripts are translated into protein and subsequently form functional α7 nAChRs.

### Technical Considerations

For this study, we utilized an *in situ* hybridization protocol that was developed not only for its sensitivity but also for specificity of signal (Winzer-Serhan et al., 1999). We employed [^35^S]UTP-labeled cRNA riboprobes transcribed from a 279 bp DNA fragment encoding the third intracellular loop of the mouse α7 nAChR subunit for increased specificity. Riboprobes targeted to this region have been used previously to characterize α7 mRNA distribution in developing mice (Orr-Urtreger et al., 1995, 2000; Broide et al., 2001), and show the same pattern of expression as that observed using riboprobes targeted to the full length mouse α7 nAChR mRNA that have been successfully used in other studies (Broide et al., 1995; Bina et al., 1995; Adams et al., 2002). BLAST analysis revealed no other highly homologous sequences to non-α7 nAChR mouse transcripts, indicating that the possibility of this cRNA probe to cross-hybridize with highly homologous sequences is very low, and we are confident that the hybridization signal shown throughout the embryonic mouse body reflect α7 mRNA expression. While the number of embryos in this study is limited to *n* = 2–3 per age, it must be emphasized that the consistency in α7 nAChR expression within the outlined structures from one age to the next provides an adequate number overall that supports our findings for developmental expression of this receptor.

Our study of α7 nAChR expression during mouse development was focused on mRNA expression and not protein. This was due to the fact that at the early prenatal time points, the autoradiographic signal for [^125^I]α-BTX binding to α7 receptor proteins in neuronal areas was barely detectable above background. Furthermore, in the periphery, [^125^I]α-BTX binds to muscle type nAChRs which makes it difficult to distinguish binding to α7 receptors from binding to muscle-type nAChRs. In addition, the use of α7 nAChR subunit antibodies has proven unreliable because of the non-specific binding to unknown sites that persist in α7 nAChR subunit knockout mice (Moser et al., 2007). For this reason, publications describing expression of α7 nAChRs based on antibody staining or Western blotting, are not discussed unless antibody specificity was verified in α7 knockout mice. Therefore, in this study, we focused our analysis on α7 subunit mRNA expression, and are conscious of the fact that while α7 mRNA expression was observed within the areas described herein, this expression may not translate into subunit protein expression and formation of functional α7 nAChRs. However, studies in the chick (Yu and Role, 1998; Shoop et al., 1999; McNerney et al., 2000), rodent (Ospina et al., 1998; Adams et al., 2002; Tribollet et al., 2004) and human (Falk et al., 2002, 2003) have demonstrated expression of α7 nAChR binding sites in the CNS and PNS during embryonic development. Nevertheless, the existence of functional α7 nAChRs in areas of low α7 mRNA expression, and in particular, in non-neuronal tissue, is still unknown, and this issue was not addressed in the current study.

### Developmental Expression in Neuronal Tissue

#### Central Nervous System and Spinal Cord

As early as E13, α7 mRNA expression was strong in the telencephalon, in particular, within the cortical and hippocampal anlage. This is in agreement with a previous study reporting that α-BTX binding can first be detected in murine hippocampus at E13 (Adams, 2003). Furthermore, a similar temporal and spatial expression pattern has been described in rat hippocampus (Adams et al., 2002). Thus, the results from this and other studies suggest that α7 mRNA is translated into α7 nAChRs at an early age, during embryonic cortical and hippocampal development, but it is yet unclear if α7 nAChRs are functional in these immature cortical structures. However, α7 nAChRs have been implicated in hippocampal excitatory synapse formation, suggesting a functional role for α7 nAChRs in the developing hippocampus (Lozada et al., 2012), and there is evidence that α7 nAChRs regulate GABAA receptor function and the developmental GABAergic switch from excitation to inhibition in ganglionic and hippocampal neurons (Liu et al., 2007).

In the spinal cord, another area of robust embryonic α7 nAChR expression, we found mRNA as early as E13, the youngest time-point examined. A previous study had detected α-BTX binding sites at E16 within rat spinal cord suggesting the presence of α7 nAChRs (Tribollet et al., 2004). Furthermore, α7 mRNA expression has been detected in human spinal cord derived from 4 to 12-week-old embryos (Hellström-Lindahl et al., 1998). Moreover, there is experimental evidence that spinal α7 nAChRs are functional and facilitate excitatory neuronal transmission in rat neonates (Genzen and McGehee, 2003; Cordero-Erausquin et al., 2004). Thus, α7 nAChRs are expressed early in the embryonic rodent and human spinal cord and may be involved in the refinement of spinal cord circuits.

#### Peripheral Nervous System

The PNS serves as an essential relay between the brain and spinal cord, and the rest of the body. Acetylcholine is the major neurotransmitter in the PNS activating muscarinic and nicotinic AChRs in different circuits. In the PNS, the main nAChR subtype is composed of α3β4 subunits, and lack of this heteromeric nAChR results in early postnatal death due to dysregulation of vital body function (Xu et al., 1999; Wang et al., 2003). In contrast, the role of α7 nAChRs in the PNS is not well defined. In this study, we detected α7 mRNA expression in dorsal root ganglia, sympathetic and parasympathetic ganglia, and in the enteric plexus starting at E13, with intensity of expression greatly increasing thereafter. Neonatal expression of α7 nAChR transcripts has also been reported in mouse superior cervical ganglion (Putz et al., 2008). Autoradiographic analysis has shown I^125^-α-BTX binding to dorsal root ganglia, superior cervical ganglion and sphenopalatine ganglion at E16 (Tribollet et al., 2004). Together these data suggest the presence of α7 nAChRs in the PNS during embryonic development. Functional α7 nAChRs have been described in dorsal root ganglia (Genzen et al., 2001; Smith et al., 2013) and different ganglionic preparations (Cuevas and Berg, 1998; Yu and Role, 1998; Cuevas et al., 2000; Li et al., 2009). In the embryonic avian ciliary ganglion, neuronal responses to α7 nAChR activation result in changes of intracellular calcium, which has been suggested to play a role in cell survival within the developing autonomic nervous system (Hruska and Nishi, 2007) and may help in synchronizing transmission in the developing ganglion (Chang and Berg, 1999). Furthermore, results from embryonic chick sympathetic neurons suggest the possibility that α7 participates in forming functional heteromeric nAChRs (Yu and Role, 1998). Heteromeric α7β2 nAChRs have since been detected in the brain but may also exist in the PNS (Wu et al., 2016). Altogether, there is strong expression of α7 mRNA and binding site, and ample evidence for α7 nAChR mediated responses in the PNS. However, the functional roles of α7 nACHRs in the PNS during development and in adults are still not clearly defined.

### Expression in the Enteric Nervous System

Neuronal nAChRs are essential for fast excitatory neurotransmission in the ENS, and the predominant nAChR subtype mediating the synaptic transmission in the myenteric plexus is the α3β4(α5) heteromeric nAChR ion channel (Galligan and North, 2004). Functional responses mediated by heteromeric nAChRs can be detected in the embryonic ENS at E12.5, and greatly increase in intensity with age (Foong et al., 2015). In contrast, the contributions of α7 nAChRs to ENS functions remains ill defined, although, a potential role for α7 nAChRs as modulator of transmitter release has been proposed (Obaid et al., 2005). In the current study, transcripts for α7 nAChR subunits were detected in the embryonic mouse gut starting at E13 and increasing in intensity shortly thereafter. Similarly, in rat myenteric plexus, expression of α7 nAChR subunit mRNA has been detected in neonates (Garza et al., 2009). However, further studies are needed to address the functional role of α7 nAChRs in the mature and developing ENS.

### Developmental Expression in Non-neuronal Tissue

In mammals, neuroendocrine chromaffin cells are located in the medulla of the adrenal glands, which is innervated by the sympathetic splanchnic nerve. Release of acetylcholine triggers the secretion of epinephrine and norepinephrine from the adrenal gland via activation of heteromeric α3β4^*^ nAChRs (Yokotani et al., 2002). Although, α7 nAChRs have been detected in the adrenal medulla, these receptors may not be involved in catecholamine release but exert other functions (Mousavi et al., 2001; Di Angelantonio et al., 2003; Sala et al., 2008; Criado, 2018). In the present study, we detected α7 transcripts in the embryonic adrenal medulla as early as E13, increasing in intensity thereafter. Our results are in agreement with another study using green fluorescent protein (GFP)-tagged α7 subunit expression demonstrating that GFP-immuno-reactivity can be detected in embryonic precursor cells of the adrenal gland as early as E12.5, and in chromaffin cells co-expressed with tyrosine hydroxylase by E18.5 (Gahring et al., 2014). Yet, the role of α7 nAChR in the embryonic adrenal gland is not well understood. However, a functional role for α7 nAChRs during prenatal development can be inferred from a chronic gestational nicotine exposure study where alterations in oxygen sensitivity of adrenal chromaffin cells depended on α7 nAChRs (Nurse et al., 2009). There is also evidence that perinatal nicotine exposure alters KATP channel function in chromaffin cells of the medulla through α7 nAChRs (Buttigieg et al., 2009). Thus, further studies are needed to verify the presence of functional α7 nAChRs in the developing and mature adrenal gland.

The neuroendocrine chromaffin cells (or pheochromocytes) of the adrenal medulla are derived from the neural crest and are related to neurons in the peripheral and enteric nervous system. Neural crest cells are also the precursor for a diverse group of non-neuronal cells. For example, cranial neural crest cells form the craniofacial mesenchyme which differentiates not only into cranial ganglia but also into craniofacial cartilage and bone (Selleck et al., 1993). Neural crest cells contribute to thymus, bones of the middle ear and jaw, and odontoblasts of the tooth primordia. Other neural crest cells are located in the genital glands, the epicardium, and in and around the kidneys. Interestingly, in the mouse embryo, low levels of α7 mRNA expression were detected in cells located within several of these tissues, including tongue, tooth bud, kidney cortex, aorta, prostate and muscle tissue, which are all related to the neural crest. In support of our findings, other studies have also reported expression of α7 during tooth development (Rogers and Gahring, 2012), in fetal muscle tissue (Fischer et al., 1999), and in a subset of circumvallate taste bud cells in rat tongue tissue (Qian et al., 2018). However, it is mostly unclear whether α7 nAChR mRNA is translated into subunit protein and subsequently into functional receptors. It is possible that α7 mRNA expression is only transient in many non-neuronal cell types, as seen in muscle tissue, and that these low levels of mRNA do not actually translate into functional receptors. However, in support of functional receptors despite low level of mRNA expression, there is evidence that α7 nAChRs contribute to proper tooth development since knockout of α7 nAChRs results in altered adult mandibular incisors morphology (Rogers and Gahring, 2012).

### Lack of α7 Transcript Detection

In contrast to α7 mRNA hybridization signal found in kidney, transcripts for α7 where not detected in other organs such as heart (except for the expression in ganglia), liver, lung, spleen and thymus. Our findings are in general agreement with a recent study compiling tissue-specific expression patterns of α7 nAChR subunit in mouse tissues. Based on the Mouse ENCODE transcriptome data set, expression of α7 nAChR mRNA in heart, liver, spleen and thymus is extremely limited, with low level of expression detected in adult kidney, lung, and testis in mice (Yue et al., 2014). Similarly, low level of α7 mRNA expression was detected in human peripheral organs, although, more widespread α7 protein expression was reported^1^.

Our data show a lack of α7 mRNA expression in thymus, liver, spleen, and lung, with some expression found in the heart that could be derived from intracardiac ganglia, and are in contrast with others reporting expression of α7 nAChR mRNA in heart (Dvorakova et al., 2005), lung (Sekhon et al., 1999), and developing mouse thymus (Kuo et al., 2002). The reasons for these discrepancies are not immediately clear, but may involve different detection approaches. Several studies used RT-PCR analysis (Kuo et al., 2002; Dvorakova et al., 2005). It cannot be ruled out that mRNA derived from neurons in ganglia embedded in organ tissues, or blood cells like macrophages, contributed to amplification of α7 mRNA transcripts, or that alternative splice variants are expressed which were not detected with our 279 bp probe (Severance et al., 2004).

We expected to detect α7 nAChR mRNA expression in lung, because there is evidence for functional α7 nAChRs in lung bronchial epithelial cells (Wang et al., 2001; Fu et al., 2003, 2009), and prenatal nicotine exposure affects lung function through α7 nicotinic receptors (Wongtrakool et al., 2012). A recent study used a reporter mouse line which indirectly detected expression of α7 nAChR subunits in mouse lung tissue (Gahring et al., 2017). However, we did not detect hybridization signal in lung tissue, which may reflect very low levels of expression in fetal and neonatal lung.

Also puzzling was the lack of expression in the thymus and spleen, organs that are related to the immune system. The α7 nAChR plays a critical role in the cholinergic anti-inflammatory pathway (Wang et al., 2003), and α7 expression has been found in immune cells including lymphocytes and macrophages (Skok et al., 2007; Fujii et al., 2017). A recent study demonstrated expression in macrophages from α7 wildtype but not knockout mice (Garg and Loring, 2019). RT-PCR technology is a highly sensitive approach that detects even minute numbers of transcripts that may go undetected with the isotopic *in situ* hybridization approach. An alternative explanation could be the age of the animals. Adult mice may exhibit higher levels of α7 nAChRs expression in immune system related tissues than fetal or neonatal tissues. However, expression of α7 nAChR mRNA in spleen and thymus is also very limited in adult mice (Yue et al., 2014). It is also possible that the differentiated state of immune cells isolated from blood affects expression of α7 mRNA, or that different splice variants of α7 transcripts are expressed in circulating immune cells. However, this conundrum still needs to be addressed, especially with the increased focus on of α7 role in cholinergic anti-inflammatory pathway.

## Conclusion

The current study focused on α7 nAChR mRNA expression in embryonic and neonatal tissues. The strongest expression of α7 mRNA was detected in the PNS, including dorsal root ganglia, parasympathetic and sympathetic ganglia, followed by strong expression in brain and spinal cord. In these structures, expression started at E13 and increased in intensity thereafter. This neuronal expression pattern generally corresponds to the distribution of alpha-BTX binding to α7 homomeric nAChRs, as demonstrated by other studies, suggesting the presence of α7 receptors, and a functional role in the PNS, brain and spinal cord during embryonic development. In addition, several reports describe physiologically active α7 nAChRs in embryonic chick ganglion neurons, (Yu and Role, 1998; Shoop et al., 1999; McNerney et al., 2000). Furthermore, there is evidence that α7 nAChRs regulate GABAA receptor function and the developmental GABAergic switch from excitation to inhibition in ganglionic and hippocampal neurons (Liu et al., 2007; Lozada et al., 2012). Thus, nAChRs are expressed early during the development of the peripheral and central nervous systems and may guide important neurodevelopmental processes. Little is known about α7 nAChRs in non-neuronal tissue during development. Although α7 mRNA expression was detected, it remains to be seen if functional receptors are present in perinatal animals. There is a growing number of studies describe anti-inflammatory effects mediated by α7 nAChRs, however, expression of α7 mRNA in immune system-related tissues during embryonic development was below the sensitivity of the *in situ* hybridization approach used in this study. Future functional studies need to determine their role in the developing immune system. Altogether, the presence of α7 nAChR mRNA within the regions described herein may likewise indicate the concomitant existence of protein, albeit at lower abundance outside of the nervous system.

## Data Availability

All datasets generated for this study are included in the manuscript and/or the supplementary files.

## Ethics Statement

This study was approved by the Institutional Animal Care and Use Committee at the University of California, Irvine, CA, United States.

## Author Contributions

RB analyzed the results, prepared the figures, drafted the manuscript. UW-S analyzed the results and wrote the manuscript. YC conducted the experiments. FL planned and oversaw the project.

## Conflict of Interest Statement

RB is employed by the company Allergan plc. The remaining authors declare that the research was conducted in the absence of any commercial or financial relationships that could be construed as a potential conflict of interest.

## Footnotes

1 https://www.proteinatlas.org/ENSG00000175344-CHRNα7/tissue
